# The Prevention of Abscesses in Hypodermic Medication

**Published:** 1871-12

**Authors:** 


*The Prevention of Abcesses in Hypodermic Medication.* By Reuben A. Vance, M. D.

The author mentions the following circumstances of importance in hypodermic medication. The solution should not be too dilute, nor too strongly acid. That the material of the needle should be of gold, and that the size of the syringe should be small, so that it may be held with ease between the fingers. He also describes the method of manipulation, and gives a discription of an instrument for the injection of strychnia.

